# 

**Published:** 1911-06-17

**Authors:** 


### MEDICAL APPOINTMENTS.

The following appointments have been made: At the Manchester Royal Infirmary: Senior House Surgeons, Mr. A. E. Woodall, M.Sc., M.B., Ch.B.Vict.; Mr. T. T. Higgins, M.B., Ch.B.Vict., M.R.C.S., L.R.C.P. Junior House Surgeon, Mr. J. R. Rigg, M.B., Ch.B.Vict. House Surgeon to Special Departments, Mr. James Cowan, M.R.C.S., L.R.C.P. House Physician, Mr. J. B. Holmes, L.S.A.London. Accident Room House Surgeon, Mr. G. J. French, M.R.C.S., L.R.C.P. Re-appointment: Mr. W. R. Douglas, F.R.C.S.Eng., Assistant Surgical Officer.

The following appointment of three additional members to the medical staff has been made at the Royal Victoria Hospital, Belfast: Assistant physician for hæmatology and vaccine therapy, Dr. Thomas Houston; assistant physician in charge of the electrical department, Dr. J. C. Rankin; anæsthetist, Dr. Fielden. The three gentlemen appointed were for several years members of the auxiliary staff of the institution. The three new resident medical officers are Dr. W. A. Anderson, Dr. P. J. Gaffikin, and Dr. W. S. Haydock.

### BUILDINGS CONTEMPLATED AND IN PROGRESS.

Corbally (Downpatrick).—Mr. E. P. Nolan, C.E., has prepared a design for a dispensary and residence at Corbally. Sureties for £1,000 are required from builders tendering for the work.

Dewsbury.—Mr. Kirk is architect for the new casualty ward which has just been completed at the Dewsbury Infirmary. The cost of the work has been £1,200.

Greenwich.—A tender of £19,965, exclusive of plumbing work, has been accepted for the extension of the Miller Hospital, in Greenwich Road. Messrs. Young and Hall, of London, FF.R.I.B.A., are the architects.

Leicester.—Alterations and extensions are proposed to the Borough Asylum at a cost of £3,000. The work is under the charge of the Town Council surveyor, Mr. E. G. Mawbey.

Maesycoed.—Messrs. Thomas and Morgan, of Pontypridd, are architects for proposed works at the infirmary and workhouse.

Sevenoaks.—Mr. Ernest Pawley, of Sevenoaks, is architect for the alterations and additions to the Union Workhouse, Ide Hill, near Sevenoaks. Tenders are being advertised for.

### GIFTS.

Mr. Samuel Firth, of Leeds, has bequeathed £200 to the Dewsbury and District General Infirmary.

Mr. A. M. Singer has given £200 to the Extension Fund of the Central London Throat and Ear Hospital.

The Cordwainers' Company has given ten guineas to the Royal Hospital for Incurables, Putney Heath.

Mr. Thomas Turner, of Brighton, a director of the Swindon United Gas Company, has left £100 to the Swindon Victoria Hospital.

Mr. Vernon Kirk Armitage, of Southport, Lancs, has bequeathed £1,000 each to the Manchester Children's Hospital, Pendlebury, the Salford Royal Hospital, the Manchester Royal Eye Hospital, and £500 each to the Royal Albert Asylum, Lancaster, and the Ancoats Hospital and Ardwick and Ancoats Dispensary.

Miss Julia Winstanley, of Waterloo, Lancs, has left £100 each to the Home for Female Incurables, Liverpool, the Bootle Hospital, the Liverpool Royal Infirmary, the Children's Hospital, Myrtle-street, Liverpool, and £80 to the Liverpool Women's Hospital; also £50 each to the Liverpool Southern Hospital and to the Zenana Medical Mission.

### JOTTINGS.

A Coronation Carnival, organised entirely by the working men of the district, is to be held on Saturday, July 29, in aid of the funds of the Gravesend Hospital.

H.R.H. Princess Louise, Duchess of Argyll, has graciously consented to open the Sir Henry Tyler Wing extension of the London Homœopathic Hospital on Thursday, July 6. Applications for tickets for the ceremony should be made to the Secretary, Mr. Edward A. Attwood, at the London Homœopathic Hospital, Great Ormond Street, W.C.

According to *Mercy and Truth* an out-patient block has been added to the existing hospital at Gaza, Palestine. The new buildings form three sides of a square facing east. The north wing consists of a hall forty-five feet long by thirty broad, and fifteen feet high. It is made of reinforced concrete, the floor being of white concrete tiles, and the walls painted green. The other wing consists of a building twenty-five feet square, under which is a basement. These are given over to the dispensary and drug store. Adjoining is a bathroom and the porter's lodge, while on the other side are two small rooms devoted respectively to a laboratory and an ophthalmic room. Between the two wings is a line of buildings to be used as consulting rooms and a theatre for minor operations. The chief expense of the erection of the new block has been borne by an anonymous donor.

### APPOINTMENTS VACANT.

Full particulars of these vacancies can be obtained on reference to our advertisement columns :

Royal Albert Hospital, Devonport.—Assistant House Surgeon.

The Prince of Wales's General Hospital, Tottenham, N.—Honorary Surgeon.

Birmingham and Midland Eye Hospital.—Junior House Surgeon.

Leeds General Infirmary.—Ophthalmic House Surgeon.

Carmarthenshire Infirmary.—Resident Medical Officer.

Wolverhampton and Staffordshire General Hospital.—House Surgeon.

